# Modelling for *Taenia solium* control strategies beyond 2020

**DOI:** 10.2471/BLT.19.238485

**Published:** 2020-01-27

**Authors:** Matthew A Dixon, Uffe C Braae, Peter Winskill, Brecht Devleesschauwer, Chiara Trevisan, Inge Van Damme, Martin Walker, Jonathan I D Hamley, Sylvia N Ramiandrasoa, Veronika Schmidt, Sarah Gabriël, Wendy Harrison, Maria-Gloria Basáñez

**Affiliations:** a Department of Infectious Disease Epidemiology, London Centre for Neglected Tropical Disease Research and MRC Centre for Global Infectious Disease Analysis, Imperial College London, London W2 1PG, England.; b Department of Infectious Disease Epidemiology and Prevention, Statens Serum Institut, Copenhagen, Denmark.; c Department of Epidemiology and Public Health, Sciensano, Brussels, Belgium.; d Department of Biomedical Sciences, Institute of Tropical Medicine, Antwerp, Belgium.; e Department of Veterinary Public Health and Food Safety, Ghent University, Merelbeke, Belgium.; f Department of Pathobiology and Population Sciences, Royal Veterinary College, Hatfield, England.; g Service de Lutte contre les Maladies Endémiques et Négligées, Ministry of Public Health, Antananarivo, Madagascar.; h Department of Neurology, Technical University Munich, Munich, Germany.; i Schistosomiasis Control Initiative Foundation, London, England.

## Abstract

The cestode *Taenia solium* is responsible for a considerable cross-sectoral health and economic burden due to human neurocysticercosis and porcine cysticercosis. The 2012 World Health Organization (WHO) roadmap for neglected tropical diseases called for the development of a validated strategy for control of *T. solium*; however, such a strategy is not yet available. In 2019, WHO launched a global consultation aimed at refining the post-2020 targets for control of *T. solium* for a new roadmap for neglected tropical diseases. In response, two groups working on taeniasis and cysticercosis mathematical models (cystiSim and EPICYST models), together with a range of other stakeholders organized a workshop to provide technical input to the WHO consultation and develop a research plan to support efforts to achieve the post-2020 targets. The workshop led to the formation of a collaboration, CystiTeam, which aims to tackle the population biology, transmission dynamics, epidemiology and control of *T. solium* through mathematical modelling approaches. In this paper, we outline developments in *T. solium* control and in particular the use of modelling to help achieve post-2020 targets for control of *T. solium.* We discuss the steps involved in improving confidence in the predictive capacities of existing mathematical and computational models on *T. solium* transmission, including model comparison, refinement, calibration and validation. Expanding the CystiTeam partnership to other research groups and stakeholders, particularly those operating in different geographical and endemic areas, will enhance the prospects of improving the applicability of *T. solium* transmission models to inform taeniasis and cysticercosis control strategies.

## Résumé

*Taenia solium* est un cestode qui entraîne une charge intersectorielle économique et sanitaire considérable en provoquant une neurocysticercose humaine et une cysticercose porcine. La feuille de route sur les maladies tropicales négligées, publiée en 2012 par l'Organisation mondiale de la Santé (OMS), appelait à développer une stratégie de contrôle validée pour *T. solium *; cependant, cette stratégie n'est pas encore disponible à l'heure actuelle. En 2019, l'OMS a lancé une procédure de consultation mondiale visant à préciser les objectifs de contrôle de *T. solium* après 2020, afin de rédiger une nouvelle feuille de route sur les maladies tropicales négligées. Deux groupes qui travaillent sur des modèles mathématiques de taeniasis et cysticercose (modèles cystiSim et EPICYST) ainsi qu'une série d'autres intervenants ont donc organisé un atelier pour fournir une contribution technique à cette consultation et développer un programme de recherche destiné à soutenir les efforts de réalisation des objectifs ultérieurs à 2020. L'atelier a donné naissance à une collaboration, CystiTeam, qui s'intéresse à la biologie des populations, à la dynamique de transmission, à l'épidémiologie et au contrôle de *T. solium* en employant des méthodes de modélisation mathématique. Le présent document retrace l'évolution du contrôle de *T. solium*, en particulier l'usage de la modélisation pour contribuer à atteindre les objectifs d'après 2020 en la matière*.* Nous abordons les diverses étapes de renforcement de la confiance accordée aux capacités prédictives des modèles mathématiques et informatiques existants sur la transmission de *T. solium*, notamment la comparaison, l'optimisation, le calibrage et la validation des modèles. Élargir le partenariat CystiTeam en intégrant d'autres groupes de recherche et intervenants, surtout ceux opérant dans différentes zones géographiques et endémiques, accroîtra les chances d'amélioration de l'applicabilité pour les modèles de transmission de *T. solium*, et permettra ainsi d'établir des stratégies de lutte contre la taeniasis et la cysticercose.

## Resumen

El cestodo *Taenia solium* es responsable de una importante carga sanitaria y económica transversal debido a la neurocisticercosis humana y la cisticercosis porcina. En la hoja de ruta de la Organización Mundial de la Salud (OMS) de 2012 sobre las enfermedades tropicales desatendidas se solicitaba la elaboración de una estrategia validada para el control de *T. solium*; sin embargo, dicha estrategia aún no está disponible. En 2019, la OMS inició una consulta mundial destinada a perfeccionar los objetivos de control de *T. solium* aplicables a partir de 2020 con miras a elaborar una hoja de ruta nueva sobre las enfermedades tropicales desatendidas. Consecuentemente, dos grupos que trabajan en modelos matemáticos de teniasis y cisticercosis (modelos cystiSim y EPICYST), junto con un grupo de otros interesados, organizaron un seminario para contribuir técnicamente a la consulta de la OMS y elaborar un plan de investigación a fin de apoyar los esfuerzos para lograr los objetivos a partir de 2020. El seminario impulsó la formación de un equipo de colaboración, CystiTeam, para abordar la biología de la población, la dinámica de la transmisión, la epidemiología y el control de *T. solium* mediante enfoques de modelos matemáticos. En el presente documento se describen las novedades en el control de *T. solium* y, en particular, la aplicación de modelos para ayudar a lograr los objetivos a partir de 2020 sobre el control de *T. solium*. Se analizan las etapas necesarias para mejorar la confianza en las capacidades de predicción de los modelos matemáticos y computacionales existentes sobre la transmisión de *T. solium*, incluyendo la comparación, el perfeccionamiento, el ajuste y la validación de los modelos. La ampliación de la asociación CystiTeam a otros grupos de investigación e interesados, en particular los que operan en diferentes zonas geográficas y endémicas, reforzará las perspectivas de mejorar la aplicabilidad de los modelos sobre las transmisión de *T. solium* para fundamentar las estrategias de control de la teniasis y la cisticercosis.

## ملخص

تعد الدودة الشريطية الوحيدة، المسؤولة عن عبء وبائي وصحي ضخم يصيب العديد من القطاعات، وذلك بسبب داء الكيسات العصبية البشري وداء الخراجات الكيسي. دعت خارطة طريق الأمراض المدارية المهملة، التابعة لمنظمة الصحة العالمية (WHO) لعام 2012، إلى تطوير استراتيجية معتمدة للسيطرة على *الدودة الشريطية الوحيدة * ؛ إلا أن هذه الاستراتيجية ليست متاحة بعد. في عام 2019، أطلقت منظمة الصحة العالمية مشاورة عالمية تهدف إلى تحسين أهداف ما بعد عام 2020 من أجل التحكم في *الدودة الشريطية الوحيدة *، وذلك لوضع خارطة طريق جديدة للأمراض المدارية المهملة. واستجابةً لذلك، قامت مجموعتان تعملان على النماذج الرياضية لداء الشريطيات وداء الخراجات الكيسية (نماذج cystiSim وEPICYST)، بالتعاون مع مجموعة من أصحاب المصلحة الآخرين، بتنظيم ورشة عمل لتقديم معطيات فنية لمشاورة منظمة الصحة العالمية، ووضع خطة بحث لدعم الجهود المبذولة لتحقيق أهداف ما بعد عام 2020. أدت ورشة العمل إلى تشكيل لجنة يُطلق عليها CystiTeam، وهي تهدف إلى معالجة بيولوجيا السكان، وديناميكيات الانتشار، وعلم الأوبئة، والتحكم في *الدودة الشريطية الوحيدة * من خلال أساليب وضع النماذج الرياضية. نوضح في هذه الورقة تطورات التحكم في *الدودة الشريطية الوحيدة *، وبخاصة استخدام وضع النماذج للمساعدة في تحقيق أهداف ما بعد عام 2020 للتحكم في *الدودة الشريطية الوحيدة *. نحن نناقش الخطوات المتبعة لتحسين الثقة في القدرات التنبؤية للنماذج الرياضية والحاسوبية الحالية والخاصة بانتشار *الدودة الشريطية الوحيدة *، بما في ذلك مقارنة النماذج وتحسينها ومعايرتها والتحقق من صحتها. إن توسيع شراكة CystiTeam لتشمل مجموعات الأبحاث وأصحاب المصلحة الآخرين، وبخاصة هؤلاء الذين يعملون في مناطق جغرافية ومتوطنة مختلفة، سوف يرتقي باحتمالات تحسين قابلية تطبيق نماذج انتشار الدودة الشريطية الوحيدة، مما يساعد في توفير المعلومات لاستراتيجيات التحكم في داء الشريطيات وداء الخراجات الكيسية.

## 摘要

绦虫*猪带绦虫*对人类脑囊虫病和猪囊虫病造成相当大的跨部门健康和经济负担。2012 年世界卫生组织（世卫组织）关于被忽视的热带病蓝图要求制定一项有效控制*猪带绦虫*的策略；然而，这一策略尚未出台。2019 年，世卫组织发起了一项全球磋商，旨在为被忽视的热带病制定新的蓝图，完善 2020 年后控制*猪带绦虫*的目标。对此，两个研究绦虫病和囊虫病数学模型的小组（cystiSim 和 EPICYST 模型）与一系列其他利益相关者一起组织了一场研讨会，为世卫组织的磋商提供技术意见，并为支持实现 2020 年后目标制定一项研究计划。该研讨会上成立了 CystiTeam 合作小组，其目的是通过数学建模方法解决种群生物学、传播动力学、流行病学和*猪带绦虫*的控制。本文概述了*猪带绦虫*控制的发展，特别是利用建模帮助实现 2020 年后的*猪带绦虫*控制目标。我们讨论了提高现有数学和计算模型对*猪带绦虫*预测能力的可信度所涉及的步骤，包括模型比较、优化、校准和验证。将 CystiTeam 合作关系扩大到其他研究小组和利益相关者，特别是那些在不同地理区域和病区开展工作的研究小组和利益相关者，将进一步改善*猪带绦虫*传播模型的应用前景，为绦虫病和囊虫病的控制策略提供参考。

## Резюме

Цестода *Taenia solium* наносит значительный урон экономике и межотраслевому сотрудничеству в области здравоохранения, поскольку она является возбудителем нейроцистицеркоза у людей и цистицеркоза у свиней. Принятая в 2012 году дорожная карта Всемирной организации здравоохранения (ВОЗ) в отношении остающихся без внимания тропических болезней призывает к разработке проверенной стратегии по борьбе с тениозом (*T. solium)*, однако данная стратегия до сих пор не разработана. В 2019 году ВОЗ объявила о проведении глобальных консультаций с целью уточнения целей по борьбе с тениозом (*T. solium)* на период с 2020 года для разработки новой дорожной карты в отношении остающихся без внимания тропических болезней. В рамках данных мероприятий две группы, работающие над математическими моделями тениоза и цистицеркоза (модели cystiSim и EPICYST), совместно с рядом других партнеров организовали семинар с целью оказания технической поддержки для консультаций ВОЗ и разработки плана исследований для обеспечения эффективности усилий, предпринимаемых для достижения целей в период с 2020 года. В результате семинара была создана совместная научно-исследовательская группа CystiTeam, задача которой состоит в исследовании вопросов популяционной биологии, динамики распространения, эпидемиологии и борьбы с тениозом (*T. solium*) на основе математического моделирования. В этой статье авторы рассказывают о разработках в сфере борьбы с тениозом (*T. solium)* и, в частности, об использовании моделирования для достижения намеченных целей по борьбе с *T. solium* на период с 2020 года. Авторы обсуждают шаги, необходимые для повышения надежности возможностей прогнозирования имеющихся математических моделей и вычислительных методов передачи тениоза (*T. solium)*, включая сравнение, уточнение, калибровку и валидацию моделей. Включение в группу CystiTeam других исследовательских групп и партнеров из различных географических и эндемических областей позволит совершенствовать применимость моделей распространения тениоза (*T. solium*) и предоставит необходимую информацию для разработки стратегий по борьбе с тениозом и цистицеркозом.

## Introduction

Infection by the cestode *Taenia solium*, a zoonotic tapeworm, exerts a considerable health and economic burden as the cause of cysticercosis in humans and pigs in endemic countries. The most acute human health burden results from neurocysticercosis-associated epilepsy, caused by cysticerci settled in the central nervous system. This disease was responsible for about 2.8 million disability-adjusted life years (DALYs) in 2010.^1^ In the United Republic of Tanzania, the economic burden for 2012 due to neurocysticercosis-associated epilepsy, has been estimated at 5.0 million United States dollars (US$) and the burden due to porcine cysticercosis, resulting from the reduced value of infected pork, at US$ 2.8 million.^2^ In Angónia district in Mozambique, with a human population of about 330 000, these estimations were about US$ 71 000 and US$ 22 000, respectively, in 2007.^3^ These data highlight the impact of *T. solium* across human and animal health sectors. *T. solium* infection disproportionately affects smallholder and subsistence farming communities in endemic settings, where the presence of common risk factors, such as free-roaming pigs and poor sanitation, allows high levels of direct and indirect (environmental) disease transmission. A collaborative One Health approach, which addresses *T. solium* control from the human health, animal health and environmental perspectives, is therefore essential to tackle this zoonotic neglected tropical disease.

## Tackling *T. solium*

The 2012 World Health Organization (WHO) roadmap *Accelerating work to overcome the global impact of neglected tropical diseases* called for the development of a validated strategy for *T. solium* control and elimination by 2015, and for interventions to be scaled up in selected countries by 2020.^4^ Research in 2018 on the control of *T. solium* concluded that evidence on optimal interventions for control and elimination is still limited.^5^ A validated strategy has, therefore, not yet been identified and, hence, the 2015 target has not been met. Nevertheless, notable progress has been made,^5^ including an elimination trial with an intensive package over a 1-year period of interventions targeting both humans and pigs on a regional scale in northern Peru.^6^ This package included mass treatment of humans with niclosamide and pigs with oxfendazole in combination with vaccination of pigs. In addition, a more targeted ring-screening intervention, in which people within a 100-m radius of pigs found positive for cysticercosis were screened and treated for taeniasis in northern Peru, showed a significant reduction in seroincidence among pigs in the intervention village after 1 year.^7^ Other intervention approaches are ongoing, including cost–effectiveness evaluations of both control and elimination objectives in Zambia.^8^ *T. solium* control could also be integrated into existing water, sanitation and hygiene projects. In addition, low-cost intervention strategies could include health education tools for human health and agricultural professionals, as well as for local communities. One such tool is the Vicious Worm.^9^ This tool has been used in the United Republic of Tanzania and Zambia to raise awareness of *T. solium* and reduce risk behaviours, such as not using latrines, improper hygiene, cooking and free-ranging pig management practices, and not seeking health care, among health-care and agricultural professionals, and schoolchildren. Computers were used in the United Republic of Tanzania, while in Zambia, the tool was projected on the wall in schools. The tool is also available as an app on smartphones with increasing numbers of people having access to such phones in sub-Saharan Africa. Studies have demonstrated a statistically significant increase in knowledge and attitudes scores in both the United Republic of Tanzania and Zambia.^10^^,^^11^ In Peru, mechanisms for transferring intervention strategies to local communities, such as community-based reporting of pig cysticercosis to inform ring strategies, have also been explored.^12^ While this study did not identify a significant change in the seroincidence of cysticercosis in the intervention group, efforts to develop locally sustainable interventions, for example, by transferring ownership of interventions, is important.

## Modelling

Inclusion of dynamic transmission modelling in intervention trials and programmes can add considerable value by predicting the long-term effect of extending interventions beyond the immediate scope of the trial. Currently, several transmission models exist which capture, to different degrees of complexity, the transmission dynamics of *T. solium* and which can be used to assess the effect of interventions. Recent research has characterized and compared the structures of these models, and their parameters and capabilities to model particular interventions.^13^ Building on this research, formal comparisons of models are needed to understand the extent of the biological and epidemiological uncertainties associated with the life cycle of *T. solium* and its transmission properties in order to identify key unknown factors that would benefit from the collection of new data (further information available in the data repository)^14^ and ultimately to build consensus on the most suitable intervention options.^15^ Some ways to fill critical data gaps and hence improve our ability to capture baseline epidemiology in models in the absence of interventions include: better characterization of local transmission dynamics, such as force-of-infection estimates (individual rate of infection acquisition in susceptible hosts), which can be obtained from human and pig age-stratified prevalence data; risk factor analysis to improve the defining of parameters, for example, of contact rates with stages infective to humans and pigs; biological factors of adult tapeworm, for example, life span and reproductive output; and environmental factors, which will likely be highly dependent on setting, for example, egg viability studies and dispersal mechanisms to better understand the distribution of environmental contamination. Research has been conducted in Peru to understand location-specific spatial dynamics, such as clustering of cysticercosis infection in pigs around human cases of taeniasis (tapeworm infection), and seasonal drivers of transmission, which could be used to inform clear spatial and seasonal transmission models.^16^ Further data are required to support model validation of intervention strategies, particularly longitudinal data before, during and after the intervention. To effectively incorporate field data into models, serological markers for true infection need to be identified because existing serological diagnostic methods for both human and pig infection have many limitations.

## Collaboration to improve modelling

**Fig. 1:**
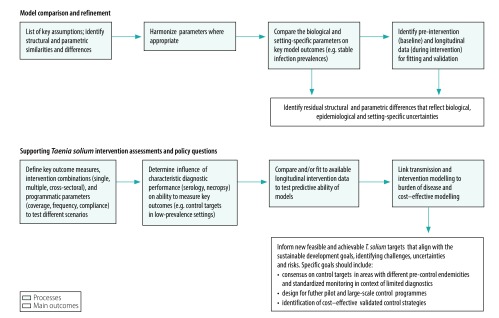
Processes and outcomes for mathematical modelling priorities to tackle *Taenia solium* control and support progress in meeting control targets

Note: This pathway is underpinned by One Health collaboration between modelling groups, field epidemiologists, programme stakeholders and policy-makers from human health, animal health and environment sectors.

Existing *T. solium* models can be improved through collaboration between modelling groups, field epidemiologists, programme stakeholders and policy-makers, and used jointly to support the design, implementation and assessment of interventions in endemic countries. To this end, a recent workshop brought together the modelling groups for the cystiSim^17^ and EPICYST^18^ models and other stakeholders to work on *T. solium* modelling (further information available in the data repository).^14^ As a result of the workshop, the CystiTeam was formed, which is a coalition of epidemiologists and programme stakeholders within the *T. solium* taeniasis and cysticercosis field. The team aims to tackle collaboratively questions on the population biology, transmission dynamics, epidemiology and control of *T. solium* through mathematical modelling approaches. Fig. 1 shows a pathway identifying *T. solium* modelling research priorities aimed at supporting progress towards *T. solium* control targets.^19^ Formal model comparison research will require initial identification of similarities and differences in key structural and parametric assumptions, which has begun to be addressed by researchers.^13^ After this step, the parameters shared between the models need to be harmonized, such as the ratio of human-to-pig population sizes, to test their influence on model outcomes, for example, stable, viable *T. solium* endemic prevalence’s. Through this process, the residual differences between the models will point to important epidemiological, biological and setting-specific uncertainties that will inform the research agenda.

With the time fast approaching for the fulfilment of the goals set in the 2012 WHO roadmap on neglected tropical diseases, attention is refocusing on defining post-2020 targets, aligned with the Sustainable Development Goals that are relevant to neglected tropical diseases.^20^ The expanding evidence base on interventions to control *T. solium* infection will provide new opportunities to define optimal, feasible and sustainable strategies that can use existing delivery platforms. At the same time, refinement of existing *T. solium* transmission models in a collaborative framework (Fig. 1) will help to re-evaluate control goals and set realistic and attainable ones. The WHO post-2020 targets for *T. solium* are based on impact indicators for the countries with intensified control in hyperendemic areas. However, technical definitions of both intensified control and hyperendemicity need clarification for progress towards these goals over the next decade to be effectively evaluated. A key modelling activity should involve predicting, in a variety of epidemiological settings, the effect of various interventions in the currently available set of tools^5^ through the different phases of a control to elimination programme.^15^

Engagement of researchers with ongoing control programmes, e.g. pilot control programme in the Antanifotsy district of Madagascar, will help evaluate the effect of interventions, both in communities that have specific taeniasis and cysticercosis interventions and in communities where anthelmintic drugs that are effective against *T. solium* are used to target other neglected tropical diseases, for example, mass administration of praziquantel for schistosomiasis. Intervention trials and control programmes will provide setting-specific data on programme parameters, such as coverage, frequency and compliance, and diagnostic performance uncertainties (Fig. 1), which will help refine modelling simulations. Integrating dynamic transmission models with burden of zoonotic disease^21^ and economic frameworks will help quantify the human health and agricultural sector gains and the cost–effectiveness of achieving newly defined global targets for zoonotic neglected tropical diseases. In northern Lao People's Democratic Republic, the cost–effectiveness of different intervention options was assessed.^22^ The interventions assessed included *T. solium* taeniasis and cysticercosis control alone and in integrated programmes that incorporated classical swine fever and soil-transmitted helminth control. Such efforts could help define model parameter values for a dynamic economic analysis in an Asian context.

Model comparison, refinement, calibration and validation lies at the heart of the CystiTeam collaboration, which aims to improve confidence in the predictive capacities of mathematical and computational transmission models and raise awareness of their usefulness. The collaboration recently provided technical input to the WHO consultation on refinement of the post-2020 neglected tropical disease goal^20^ (Box 1 and Table 1; data repository).^14^ The collaboration will bring about better engagement and dialogue between a broad spectrum of actors and stakeholders and will facilitate progress in tackling the persistent global public health and economic problems caused by *T. solium* beyond 2020. To this end, expanding this partnership to include other research groups, particularly based in Asia and Latin America, will be crucial to broaden the applicability of efforts to model *T. solium* transmission.

Box 1 **Tackling *Taenia solium* taeniasis and cysticercosis**

**WHO goals**

- validated strategy for control of *T. solium* taeniasis and cysticercosis available by 2015
- interventions scaled up in selected countries for *T. solium* taeniasis and cysticercosis control by 2020

**2030 target**

- endemic countries (*n* = 17) with intensified control in hyperendemic areas

**Is the 2030 target technically feasible with the current intervention options and tools?**

- yes, if realistic control targets are proposed
- modelling can inform the design and evaluation of pilot and large-scale control programmes with current and complementary intervention strategies^8^^,^^13^
- cystiSim and EPICYST, computational transmission models, are applicable;^17^^,^^18^ cystiSim is already in use in Zambia and Latin America, and an additional transmission model has also been developed for Latin America^23^^,^^24^

**What is required to achieve the target?**

- standardized definition of control put forward by WHO expert group
- standardized monitoring to evaluate progress of intervention strategies
- long-term intervention approaches to assess long-term epidemiological impact

**Are current tools able to reliably measure the target?**

- many limitations exist with current (serological and other) diagnostic methods^25^
- necropsy in pigs is the most reliable measure of infection, but limitations remain in assessment of long-term effectiveness of interventions (models can assist)

**What are the biggest unknowns?**

- true prevalence of *T. solium* infection in humans and pigs because of poor diagnostic methods
- adult tapeworm life span
- effect of pig-to-people population ratio on transmission
- processes regulating parasite acquisition in humans and pigs^26^
- health and economic burden^2^^,^^3^ and cost–effectiveness of interventions (DALYs likely to underestimate disease burden); possible use of the zoonotic zDALY metric^21^
- linking infection to disease models, particularly to human neurocysticercosis and epilepsy^27^

**What are the biggest risks?**

- long-term sustainability of interventions

DALY: disability-adjusted life year; WHO: World Health Organization.

**Table 1:** Priorities, applicability of modelling, data needs and timelines for informing the 2021–2030 milestones for reduction in *Taenia solium* taeniasis and cysticercosis

Priority issue identified in discussion with WHO	How can modelling address this issue?	What data are required and are they currently available?	Next steps and likely timeline
Identify risk areas where data and surveillance are lacking	geospatial mapping and modelling for environmental suitability analysis of likely endemic areas and populations at risk mapping of areas with suspected or probable coendemicity with other helminth infections that are being tackled with common preventive chemotherapy tools	proxy variables available more data needed – more detailed information in regions not yet explored; updates from other areas not available	geospatial model expected to be completed by 2021 by CystiTeam members and other collaborators (funding application submitted)
Identify the needs to start looking for potential interventions	models to predict the effect of various interventions available (e.g. cystiSim and EPICYST)	control pilot data (e.g. Madagascar^28^) longitudinal data from programmes (e.g. CystiStop, Zambia^8^) to predict temporal trends in infection during interventions data from other types of interventions	control pilot data expected to be available in 2020; longitudinal intervention data expected to be available in 2020; further data to follow work planned by CystiTeam in 2019–2021
Set thresholds for control and risk areas	inform control targets and different thresholds; need for standardized monitoring geospatial models evidence synthesis to inform policy	current models can be used with available data on diagnostic sensitivity and specificity	model comparison planned by CystiTeam for 2020
Correlate with impact of schistosomiasis MDA	adaptation of current models to simulate the added value of schistosomiasis MDA	epidemiological and programmatic data from co-endemic areas before and after MDA	adaptation of agent-based model (CystiSim) assessed with data from Zambia^8^ (2020) possible impact simulation with population-based and age-structured EPICYST model (2020)
Cost–effectiveness analysis of different interventions	adaptation of current models to explore this analysis effectiveness metrics: if DALY-based, then need to link infection model with disease model (sequelae and disability weights). DALYs likely to underestimate burden of zoonotic neglected tropical diseases cross-sectoral impact by analysing burden of disease using zoonotic (zDALYs) indicator^21^ use of WHO FERG study on global burden of disease to calculate DALYs for all parasites considered	cost–effectiveness data on various interventions and settings cost–effectiveness studies ongoing in the field key gap is link with neurocysticercosis	linking *T. solium* transmission models to burden of disease frameworks being explored by CystiTeam (2019–2021)
Best way to monitor and evaluate the impact of interventions	prevalence of cysticercosis in pigs incidence of neurocysticercosis in humans	reliable necropsy data: full carcass dissection best option for pig cysticercosis, but not always possible as requires removing animals from study areas	work planned by CystiTeam in 2019–2021

WHO: World Health Organization, MDA: mass drug administration, FERG: Foodborne disease burden Epidemiology Reference Group, DALY: disability-adjusted life year.

Note: The CystiTeam is a coalition of field and quantitative epidemiologists and programme stakeholders working on *T. solium* taeniasis and cysticercosis, which was recently formed to tackle collaboratively questions on population biology, transmission dynamics, epidemiology and control of *T. solium* through mathematical modelling approaches (list of contributors available in data repository).^14^
